# The Internet-Based Cognitive Assessment Tool: System Design and Feasibility Study

**DOI:** 10.2196/13898

**Published:** 2019-07-26

**Authors:** Pegah Hafiz, Kamilla Woznica Miskowiak, Lars Vedel Kessing, Andreas Elleby Jespersen, Kia Obenhausen, Lorant Gulyas, Katarzyna Zukowska, Jakob Eyvind Bardram

**Affiliations:** 1 Digital Health Section Department of Health Technology Technical University of Denmark Kongens Lyngby Denmark; 2 Copenhagen Center for Health Technology Technical University of Denmark Kongens Lyngby Denmark; 3 Department of Psychology University of Copenhagen Copenhagen Denmark; 4 Neurocognition and Emotion in Affective Disorders Group, Copenhagen Affective Disorder Research Centre Psychiatric Centre Copenhagen Copenhagen University Hospital Copenhagen Denmark; 5 Copenhagen Affective Disorder Research Centre Psychiatric Centre Copenhagen Copenhagen University Hospital Copenhagen Denmark

**Keywords:** screening, memory, executive function, bipolar disorder, depression, cognitive impairments, neuropsychological tests, computer software, speech recognition software

## Abstract

**Background:**

Persistent cognitive impairment is prevalent in unipolar and bipolar disorders and is associated with decreased quality of life and psychosocial dysfunction. The screen for cognitive impairment in psychiatry (SCIP) test is a validated paper-and-pencil instrument for the assessment of cognition in affective disorders. However, there is no digital cognitive screening tool for the brief and accurate assessment of cognitive impairments in this patient group.

**Objective:**

In this paper, we present the design process and feasibility study of the internet-based cognitive assessment tool (ICAT) that is designed based on the cognitive tasks of the SCIP. The aims of this feasibility study were to perform the following tasks among healthy individuals: (1) evaluate the usability of the ICAT, (2) investigate the feasibility of the ICAT as a patient-administered cognitive assessment tool, and (3) examine the performance of automatic speech recognition (ASR) for the assessment of verbal recall.

**Methods:**

The ICAT was developed in a user-centered design process. The cognitive measures of the ICAT were immediate and delayed recall, working memory, and psychomotor speed. Usability and feasibility studies were conducted separately with 2 groups of healthy individuals (N=21 and N=19, respectively). ICAT tests were available in the English and Danish languages. The participants were asked to fill in the post study system usability questionnaire (PSSUQ) upon completing the ICAT test. Verbal recall in the ICAT was assessed using ASR, and the performance evaluation criterion was word error rate (WER). A Pearson 2-tailed correlation analysis significant at the .05 level was applied to investigate the association between the SCIP and ICAT scores.

**Results:**

The overall psychometric factors of PSSUQ for both studies gave scores above 4 (out of 5). The analysis of the feasibility study revealed a moderate to strong correlation between the total scores of the SCIP and ICAT (r=0.63; *P*=.009). There were also moderate to strong correlations between the SCIP and ICAT subtests for immediate verbal recall (r=0.67; *P*=.002) and psychomotor speed (r=0.71; *P*=.001). The associations between the respective subtests for working memory, executive function, and delayed recall, however, were not statistically significant. The corresponding WER for English and Danish responses were 17.8% and 6.3%, respectively.

**Conclusions:**

The ICAT is the first digital screening instrument modified from the SCIP using Web-based technology and ASR. There was good accuracy of the ASR for verbal memory assessment. The moderate correlation between the ICAT and SCIP scores suggests that the ICAT is a valid tool for assessing cognition, although this should be confirmed in a larger study with greater statistical power. Taken together, the ICAT seems to be a valid Web-based cognitive assessment tool that, after some minor modifications and further validation, may be used to screen for cognitive impairment in clinical settings.

## Introduction

### Background

Cognitive impairment is prevalent in patients with unipolar disorder (UD) and bipolar disorder (BD) even during periods of remission, and it has a negative impact on the quality of life and psychosocial functioning. Nevertheless, cognitive function is rarely assessed in the clinical treatment of these affective disorders because of the time requirement for cognitive tests, which often exceeds the limited health care resources.

To date, there is no patient-administered tool that provides a brief and accurate screening for objective cognitive impairment using gold-standard, performance-based cognitive tasks for patients with affective disorders. The International Society for Bipolar Disorder (ISBD) Targeting Cognition Task Force recently recommended the systematic assessment of cognition in the clinical management of these patients using objective, performance-based cognitive tests [1]. However, validated tests with sensitivity to cognitive impairments in affective disorders only exist in paper-and-pencil or computerized formats, which must be administered by health care professionals. One such test for affective disorders is the screen for cognitive impairment in psychiatry (SCIP). The SCIP is a short (<15 min) paper-and-pencil test administered by trained health care professionals and comprises 5 subtests: (1) list learning (LL), (2) consonant repetition (CR), (3) verbal fluency (VF), (4) delayed list learning (DLL), and (5) visuomotor tracking (VMT) tests. These tests assess verbal recall, working memory, VF, delayed recall, and psychomotor speed, respectively [2]. The ISBD Targeting Cognition Task Force recommends the SCIP for cognitive screening in patients with BD based on recent validation studies [3,4]. In particular, studies point to the validity and reliability of the SCIP for detecting cognitive impairment in BD [5] and UD [6].

Nevertheless, even such brief screening for cognitive impairment in the clinical setting may require too much time and training of health care professionals to be realistic for all patients. This highlights the need for a patient-administered digital tool that provides a brief and valid assessment of cognition with objective cognitive tests, such as the SCIP, for affective disorders.

### Previous Studies

Our study is mainly concerned with digital cognitive test batteries, and it partly deals with the application of automatic speech recognition (ASR) in psychiatry. An overview of the related works is presented in the following 2 sections.

#### Digital Cognitive Test Batteries

In this section, validated digital tools developed for cognitive assessment are presented. Cognitive training tools are, therefore, excluded.

CANTAB Mobile [7] is a validated patient-administered tool to screen for dementia. This app examines memory impairment in patients aged 50 to 90 years using the paired associates learning test. Central nervous system vital signs (CNSVS) is a computerized neurocognitive test battery developed to evaluate cognitive impairment in mental disorders, including UD. The CNSVS has 7 tests, including verbal and visual memory, finger tapping, symbol digit coding, the Stroop test, a shifting attention test, and a continuous performance test [8]. According to the findings by Gualtieri and Johnson, CNSVS is suitable for cognitive assessment and screening of normal subjects. Another test battery is Cogstate, which is aimed to screen patients with Alzheimer’s disease but has been used to assess other neuropsychiatric disorders. A recent clinical study on Cogstate [9] aimed to examine cognitive impairment in UD patients compared with healthy controls in terms of psychomotor speed, alertness, visual memory, working memory, verbal memory, and learning and executive functions. Cogstate measures showed impairment in attention and verbal memory and learning, whereas no difference was found in psychomotor speed, visual attention, and working memory in UD patients versus controls. This contrasts with the literature on moderate impairments within these domains in UD and it could be because of the ceiling effects of the Cogstate. The THINC-it is a more recent cognitive assessment tool designed specifically for UD patients that measures attention, working memory, and executive function. This application is the first Web-based patient-administered cognitive screening tool developed for UD and thus represents an important step toward more common assessments of cognition in the clinical management of UD. The THINC-it uses gamified cognitive tasks to engage patients in taking the tests. For example, the *Trails* game is adapted from the trail-making test part B. According to the latest study [10], 100 healthy controls were tested for temporal stability and reliability as well as the validity of the THINC-it. Overall, high stability and reliability and moderate validity were found.

#### Automatic Speech Recognition in Cognitive Assessment Applications

Recently, ASR has been utilized to examine verbal impairment in mental disorders. Semantic VF as a determinant factor in mild cognitive impairment (MCI) has been automated through ASR in recent studies [11-14]. Troger et al [14] applied ASR to examine semantic VF in dementia via a telephone-based approach, showing the feasibility of automated analysis in screening for dementia. Toth et al in their recent study [13] derived nonverbal acoustic features, such as the duration of pauses, from ASR among the Hungarian population. Their findings revealed significant differences between healthy individuals and MCI patients in terms of their acoustic features of delayed recall.

#### The Gaps in the Literature

The limitations of THINC-it are twofold. First, of the cognitive domains assessed by THINC-it, only psychomotor speed shows a moderate correlation with the standardized tests. Second, THINC-it does not examine verbal memory, although this cognitive measure is a predictor of a long-term psychological functional outcome in UD and BD patients [15]. CANTAB Mobile and CNSVS do not assess verbal memory as well. The lack of verbal memory assessment might be partially because of uncertainty about how to measure it via a digital tool. Moreover, the tests suggested by CANTAB and CNSVS for use in affective disorders have not been specifically developed to screen for cognitive impairment in UD and BD patients and may thus not have optimal sensitivity for impairments in these groups.

#### Internet-Based Cognitive Assessment Tool

We developed the internet-based cognitive assessment tool (ICAT) with the perspective that it can be administered by patients themselves at home. Specifically, the ICAT is a Web-based cognitive test battery that examines immediate and delayed verbal recall, working memory, executive function, and psychomotor speed in 5 short tasks. Speech recognition technology has become advanced enough to be used in various applications. Moreover, ASR requires minimum technology and resources for remote examination. Therefore, ASR is utilized in 2 ICAT subtests to assess immediate and delayed verbal recall.

#### Goals of This Study

The objective of this paper is threefold: first, to present the ICAT as a Web-based cognitive test battery designed based on the cognitive tests included in the SCIP; second, to present 2 studies assessing 2 aspects of the ICAT—(1) its usability and (2) its feasibility evaluated by correlation analysis between the SCIP and ICAT subtests and total scores; third, to evaluate the accuracy of the ASR for immediate and delayed verbal recall.

## Methods

### Design Methods

The ICAT user interface (UI) was designed in a user-centered design process involving computer scientists, health informaticians, psychiatrists, and psychologists. Overall, the design process took 5 months and was performed in 4 consecutive stages, as explained below.

#### Phase 1: Brainstorming Design Sessions

The essential components of the ICAT system as a patient-administered system were brainstormed in 3 weekly meetings. In addition, the technical opportunities and limitations of computerizing the SCIP subtests were investigated.

#### Phase 2: Personas and User Interface Design

To identify design requirements and system functionalities, 2 personas were prepared based on the inputs received from psychiatrists and psychologists, who provided the practical lived experiences of the patients. A flowchart was created based on the personas to determine the navigation through different components (eg, homepage, instructions, and cognitive assessment tasks), and UI wireframes of each page were drawn.

#### Phase 3: Mock-Up

The wireframes were presented as a slideshow and thoroughly discussed by the ICAT team members during user experience (UX) prototyping sessions. During these sessions every aspect of the ICAT was (re)designed, including the layout and graphical design of each page, the instructions, the use of speech recognition, the feedback to the users, the use of input modalities (ie, keyboard and mouse), and the informed consent pages. During the design process, the original SCIP tasks were significantly modified for administration on Web-based technology in a browser, particularly considering support for a PC-based setup with keyboard and mouse. In this phase, the homepage of the ICAT contained a welcome page and a speaker test (see Multimedia Appendix 1).

#### Phase 4: Prototyping

The low-fidelity mock-up of the ICAT was gradually turned into a functional prototype using Web technology for graphical rendering in a browser but with no storage or persistence. This prototype was used for the initial assessment during UX prototyping sessions involving PH, KWM, LVK, and JEB. The slideshows created during phase 3 were expanded to 4 pages in the low-fidelity mock-ups (see Multimedia Appendix 1); the first page was added to determine how the patient would be notified to take the test, and the fourth page was the consent form. The final prototype was used to deploy the ICAT application on a Web platform.

### System Description

The ICAT includes the following 3 overall sections, which are presented one after another to the user: (1) the homepage, including an introduction, general instructions, and an informed consent form; (2) the technical setup (speaker and microphone test), and (3) cognitive assessment tasks. The ICAT supports both English and Danish, and users can hence select their preferred (native) language before proceeding to the general instructions. For readability, the lengthy instructions were divided into multiple pages. The terms of use in the consent form clarifies the purpose of the study, what data are gathered, and how the user’s data will be handled. All of this complies with the European data protection law (general data protection regulation, GDPR). As the ICAT makes extensive use of ASR, the second section (technical setup) ensures that the microphone and speakers are properly configured. See Multimedia Appendix 1 to check the final design of the ICAT homepage and technical setup, respectively. The third section of the ICAT contains a set of 5 short tasks, each including a test introduction and task-specific instructions. These 5 tasks were modified versions of the following:

- SCIP LL
- SCIP CR
- Wechsler Adult Intelligence Scale letter-number sequencing (WAIS LNS)
- SCIP DLL
- SCIP VMT

All of the ICAT subtests were adapted from the SCIP except for the third subtest that was replaced with a modified version of WAIS LNS. A detailed description of each ICAT task can be found in Table 1. The ICAT WAIS LNS and VMT subtests present a practice set to the users beforehand. The practice sets were adapted from their corresponding clinically administered tests. In total, the 5 tasks of the ICAT take 20 to 30 min to complete.

**Table 1:** Description of the internet-based cognitive assessment tool subtests.

Task features	Task 1: list learning^a^	Task 2: consonant repetition^b^	Task 3: Wechsler Adult Intelligence Scale letter-number sequencing^c^	Task 4: delayed list learning^d^	Task 5: visuomotor tracking^e^
Measure	Verbal memory (immediate recall)	Working memory	Working memory	Delayed verbal memory (delayed recall)	Psychomotor speed
Scoring criteria	Total number of correctly recalled words for 3 trials	Total number of correctly recalled letters	Total number of correctly sorted sequences	Total number of correctly recalled words	Total number of correct matching letters
Score range	0–30	0–24	0–21	0–10	0–30
Practice test	No	No	Yes	No	Yes

^a^An audio file containing a list of 10 words is played to the patient. Following that, the patient recalls as many words as possible and speak them aloud. This task is repeated 2 more times (3 trials in total) using the same word list.

^b^First, a sequence of letters is played via an audio file. Then, the patient should sort a set of numbers in descending order within a certain time period (this task is only for delaying the response). After time is up, the patient recalls and types the letters that were read to him or her earlier.

^c^A set of letter-number sequences are displayed on the screen one by one. Each sequence is played via an audio file to the patient. Following that, the patient sorts the numbers and letters of the sequence and types them.

^d^In this task, the patient should recall the same words that were played in the first list learning task and speak them aloud. No audio is played for the patient in this task.

^e^A table including 6 letters and their matching codes (a combination of circles and asterisks) is shown to the patient. In 30 seconds, the patient enters the matching letters of 30 random codes one by one.

### Modified Elements of the Screen for Cognitive Impairment in Psychiatry Tasks

#### List Learning and Delayed List Learning Tasks: Utilizing Speech Recognition

**Figure 1:**
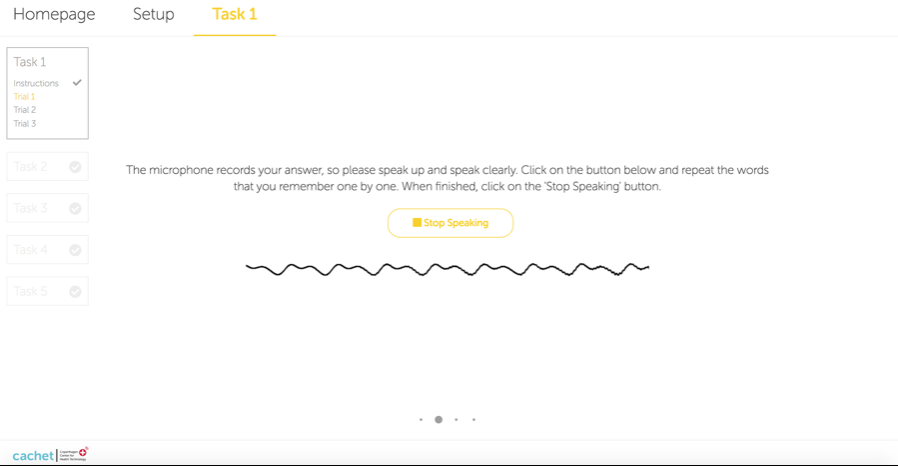
Screenshot of a sound wave received from a user’s microphone device during a recall phase of the internet-based cognitive assessment tool list learning task.

**Figure 2:**
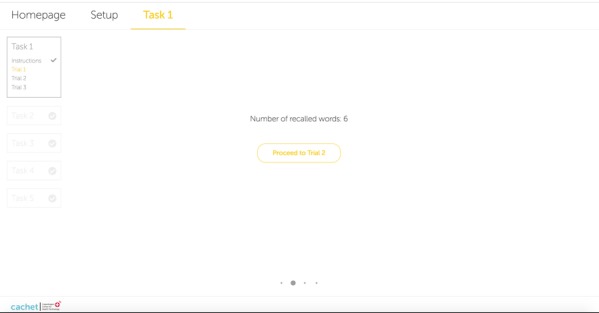
Screenshot of the number of recalled words recognized by automatic speech recognition when the user stops speaking in the list learning task.

During the initial design of the ICAT LL and DLL subtests, users were supposed to type the recalled words. However, typing was not a suitable input technique for 3 reasons. First, typing influences human visual short-term memory that may help the users in practicing the words. Hence, practicing could significantly increase the users’ scores in the second and third trials of the LL task. Second, typing skill depends on the people’s age and previous typing experience. Third, misspelled words may cause a problem when scores are automatically calculated. To clarify the latter, the SCIP administrator reads the words aloud and gives scores based on what he or she hears from the patient. Hence, giving a score to a misspelled word is unclear. An editing option for the ASR transcript could allow users to check and modify it after a recall phase. However, this approach would display the words to the users, which would then significantly improve their verbal scores because (1) all trials of the LL subtest use the same set of words and (2) it would not comply with the SCIP administration manual. By considering these major issues, the alternative to typing was to utilize ASR. Figure 1 shows the UI of the ICAT LL subtest including a user’s sound wave received from the microphone device during a recall phase. Figure 2 displays the number of recalled words calculated based on the real-time ASR when the user stops speaking. The ICAT DLL task has an interface and functionality similar to the LL task except that no audio file is played for the users.

#### Consonant Repetition Task: Sorting Numbers Using Drag-and-Drop

**Figure 3:**
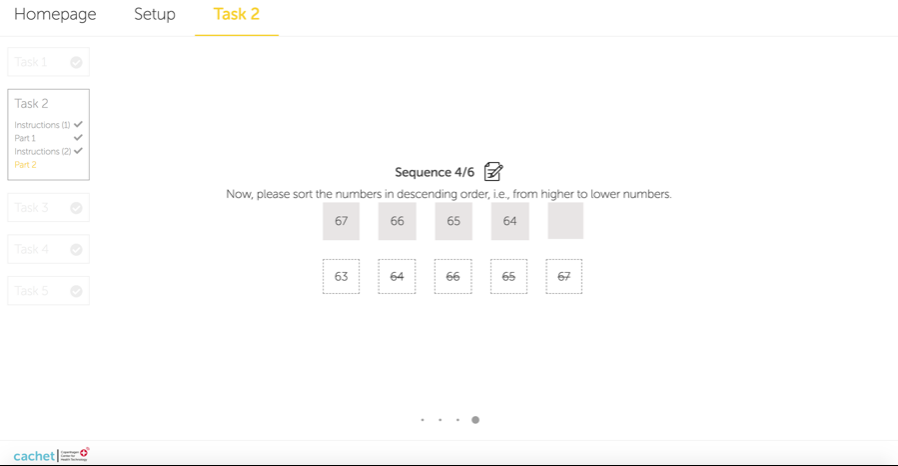
Screenshot of the internet-based cognitive assessment tool consonant repetition task where the user should sort the numbers in descending order by dragging and dropping the numbers into their correct place.

During the SCIP CR task, the test administrator asks the patient to count backwards by starting from a specific number for a time period. We replaced this face-to-face countdown with a sorting module in the ICAT CR subtest, where the users should drag each number and drop it into its correct place. The numbers displayed on the user’s screen should be placed in descending order. Figure 3 shows a sample drag-and-drop task where users should sort a sequence of numbers from 67 (highest) to 63 (lowest) within a certain time limit. Each sequence includes 5 numbers, and if the user sorts them correctly, the next set automatically appears on the screen.

#### Wechsler Adult Intelligence Scale Letter-Number Sequencing Task: Replacing Verbal Fluency

**Figure 4:**
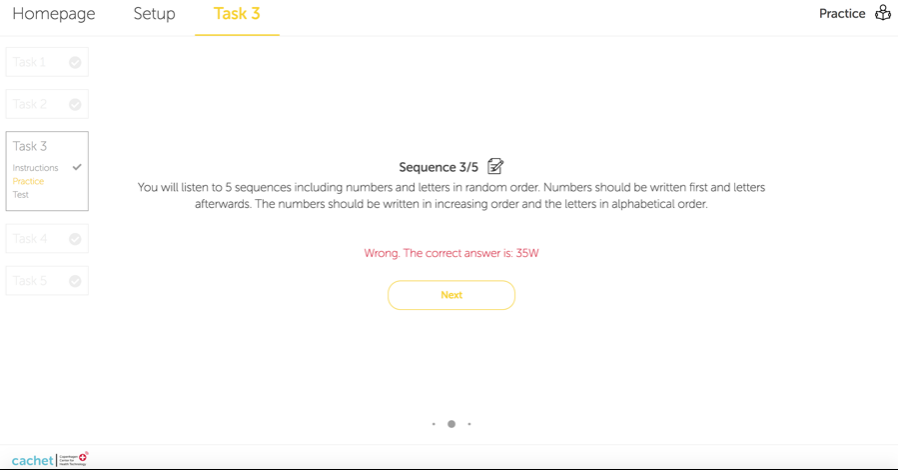
The internet-based cognitive assessment tool Wechsler Adult Intelligence Scale letter-number sequencing task includes a practice set with 5 sequences to prepare the user for the actual test. This screenshot shows that a user sorted a sequence incorrectly.

The SCIP subtest for the assessment of the VF requires the patient to generate as many words as possible that start with a specific letter, for example, *F* in 30 seconds. The third subtest of the ICAT uses WAIS LNS because the SCIP VF task could not be implemented adequately in the technology. Hence, VF was replaced with WAIS LNS, which measures executive function. Figure 4 shows an example of an incorrect response to a stimulus during a practice test of the ICAT WAIS LNS subtest.

**Figure 5:**
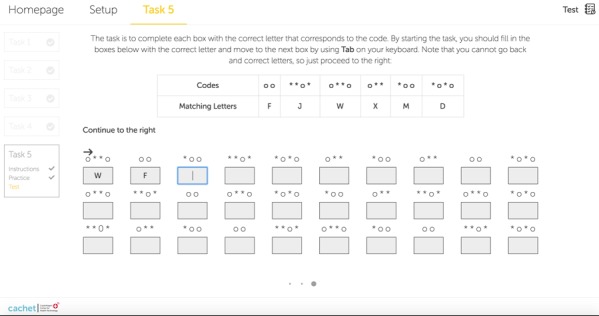
The user interface of the internet-based cognitive assessment tool visuomotor tracking task, where the user should enter the matching letter for each symbol as fast as possible.

#### Visuomotor Tracking Task: Changing Morse Codes

A table of 6 letters and their corresponding codes is written for the patients during the test, and they are required to write down the matching Morse code of 30 letters on a paper within 30 seconds. Owing to slow typing, especially among elderly people, we decided to ask users to enter the matching letter of each code in the ICAT VMT task. The Morse codes of the SCIP VMT task were modified from dots and dashes into a different combination of circles and asterisk symbols because of the learning effect for those participants who are already familiar with the Morse codes. The earlier design of this task can be found in our previous publication [16]. According to the former design of this task, a countdown clock was displayed to the user during the test, but it was later removed to prevent distraction. Figure 5 shows the current design of the ICAT VMT task.

### Technical Specifications and Apparatus

The low-fidelity mock-up of the ICAT was created in the Balsamiq desktop app [17]. The front end of the ICAT was built using React (version 15.4.0) developed by Facebook incorporation company. The Copenhagen Center for Health Technology—CACHET Research Platform (CARP), which implemented an open mobile health (mHealth) data storage unit [18], was used as the data back end, and ICAT-specific JavaScript object notation (JSON) schemas for the cognitive functions were designed according to the open mHealth specifications. Google’s ASR service [19] was used in the LL and DLL subtests, which require Google Chrome to run the application. CARP and the ICAT system are deployed on secure servers at the Technical University of Denmark. For the evaluation and feasibility studies, ICAT tests were administered using a MacBook Pro (Retina 15 inch) laptop and an external mouse for those who were not comfortable with the MacBook touchpad. Pearson correlation analysis was performed in SPSS.

### Usability and Feasibility Studies

The local ethics committee for the Mental Health Services, Capital Region of Denmark, determined that their permission for the study was not needed because it involved no testing of biomedical products nor involved any invasive procedures. A total of 2 studies were conducted: the first study was a usability test, which we will refer to as Study 1, and the second is a feasibility study, which will be called Study 2 in the rest of this paper. Participants of both studies signed an informed consent before the data collection. The informed consent was compliant with the GDPR regulation to protect the personal data of the users. In the following sections, we elaborate on the participants and procedures of the studies individually.

#### Participants

All participants were healthy individuals. Study 1 included healthy students and individuals from the campus of the Technical University of Denmark and the city of Copenhagen. The inclusion criterion was English or Danish language skills, and the exclusion criterion was any hearing disability because some of the ICAT tasks used audio files. Study 2 included healthy participants who were recruited from blood banks at hospitals within the Capital Region.

#### Procedure

The age and gender of the subjects were collected before conducting both studies. Study 1 was conducted during June and August 2018. The study leader (PH) first asked the native language of the participant. Then, PH introduced the ICAT system to the participant and briefly explained the purpose of the study. The think-aloud method [20] was used during the test. The participants were not supposed to receive assistance during the test except for login issues. Study 2 was conducted during August and September 2018. Each participant first performed the Danish version of the SCIP (SCIP-D) as administered by research assistants in the Neurocognition and Emotion in Affective Disorders group (AEJ, KO) and then completed the ICAT test.

The usability of the ICAT UI was evaluated in both studies by the poststudy system usability questionnaire (PSSUQ) [21]. Upon completing the ICAT test, the PSSUQ questionnaire was sent to the subjects’ email via Google Form, and the study leaders conducted a brief follow-up interview with the participant. During the interview, the participants were asked to mention any general or task-specific issues or suggestions. Participants could also type further comments at the end of the PSSUQ form. The voice of the users was recorded during the ICAT test and the follow-up interviews. The manually generated transcripts of the participants’ verbal responses during the ICAT LL and DLL subtests were obtained from their recorded files.

### Metrics

#### Usability Factors

PSSUQ includes 19 items, each rated on a 5-point Likert-type scale ranging from 1 (strongly disagree) to 5 (strongly agree). The psychometric factors of the PSSUQ are (1) overall usability, (2) system usefulness, (3) information quality, and (4) interface quality.

#### Word Error Rate

Previous studies used word error rate (WER) as the performance measure of ASR [11,12,14]. If N is the total number of words, D is the number of deletions, S is the number of substitutions, and I is the number of insertions, then, WER = (S+D+I)/N. WER is calculated by comparing ASR transcripts to the manually generated transcripts for English and Danish responses during the ICAT LL and DLL subtests.

#### Correlation Analysis

Pearson 2-tailed correlation analysis was performed at the .05 significance level for both the SCIP and ICAT subscores and total scores of the participants of Study 2.

#### Data Exclusion

The ICAT data of the WAIS LNS subtest were lost for 3 participants of Study 2. The correlation analysis was, therefore, performed for 16 participants.

## Results

### User Statistics

Study 1 included N=21 subjects—9 females and 12 males, with an average age of 31 years (SD 12). Of the Danish-speaking participants, 7 were native Danish speakers and 2 were citizens of Copenhagen who had spoken Danish for at least 10 years. As for the rest of the participants, 1 was a native English speaker and 11 spoke other languages. Study 2 included N=19 subjects—13 females and 6 males, with an average age of 36 years (SD 15). All participants of this study had Danish as their native language.

### Internet-Based Cognitive Assessment Tool Test Scores

**Figure 6:**
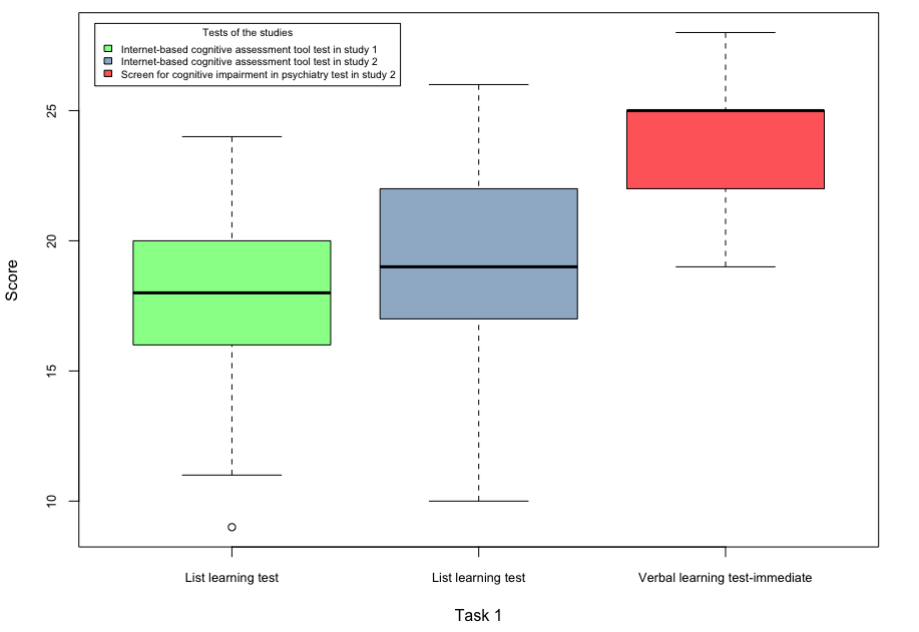
Boxplots of the internet-based cognitive assessment tool and screen for cognitive impairment in psychiatry subscores of the participants of both studies in task 1.

**Figure 7:**
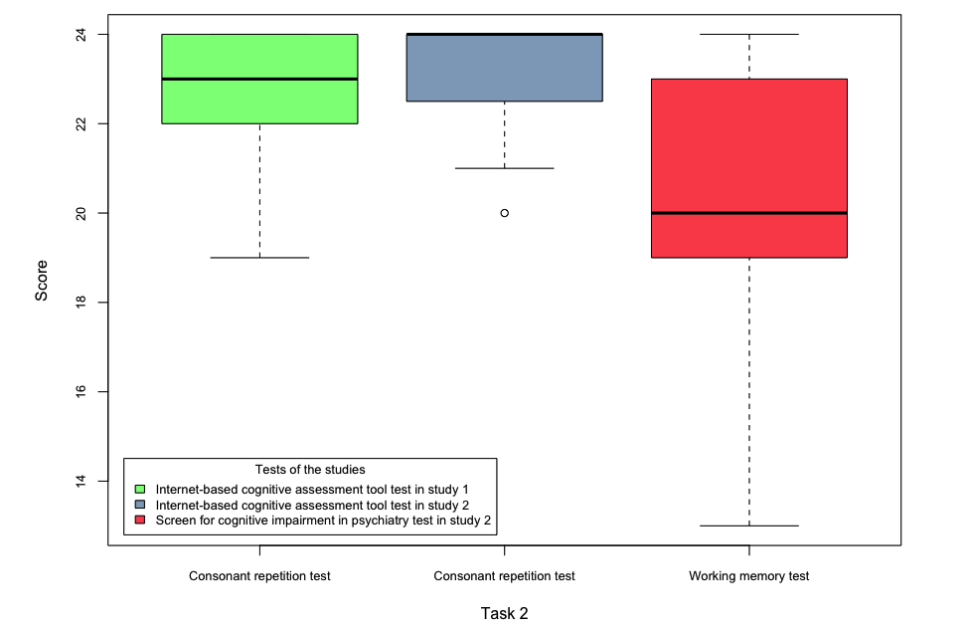
Boxplots of the internet-based cognitive assessment tool and screen for cognitive impairment in psychiatry subscores of the participants of both studies in task 2.

**Figure 8:**
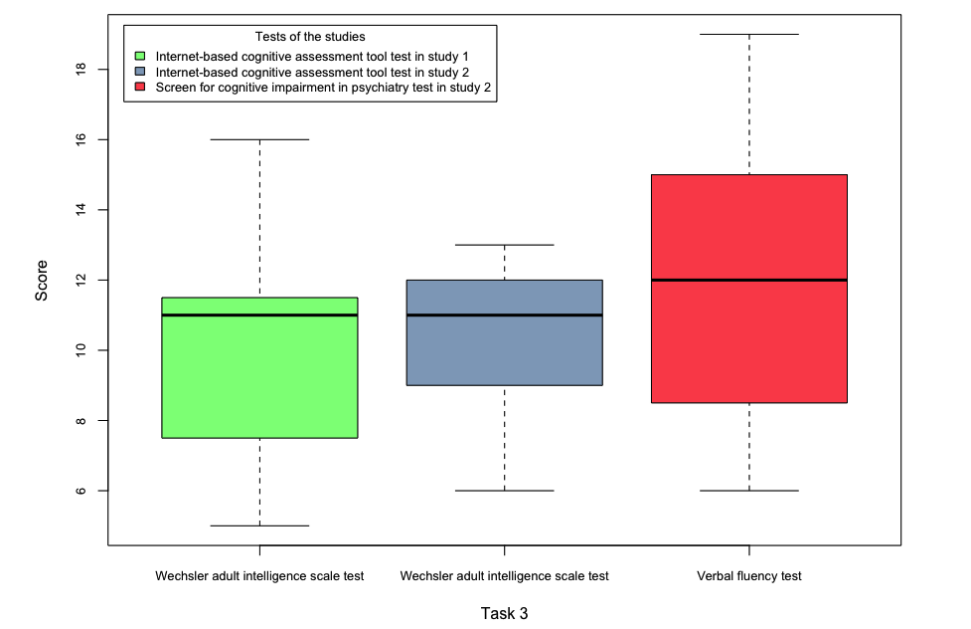
Boxplots of the internet-based cognitive assessment tool and screen for cognitive impairment in psychiatry subscores of the participants of both studies in task 3.

**Figure 9:**
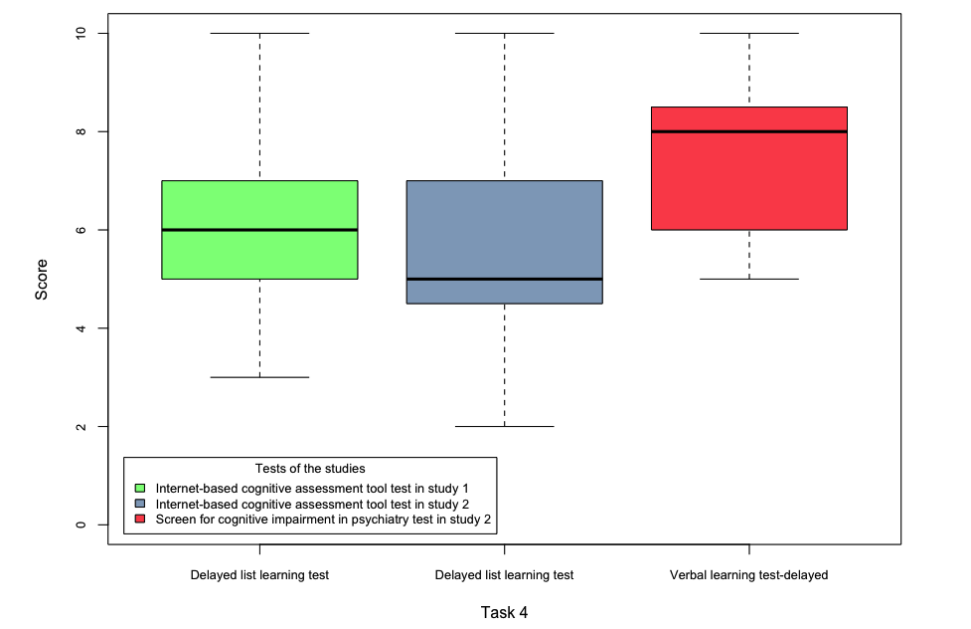
Boxplots of the internet-based cognitive assessment tool and screen for cognitive impairment in psychiatry subscores of the participants of both studies in task 4.

**Figure 10:**
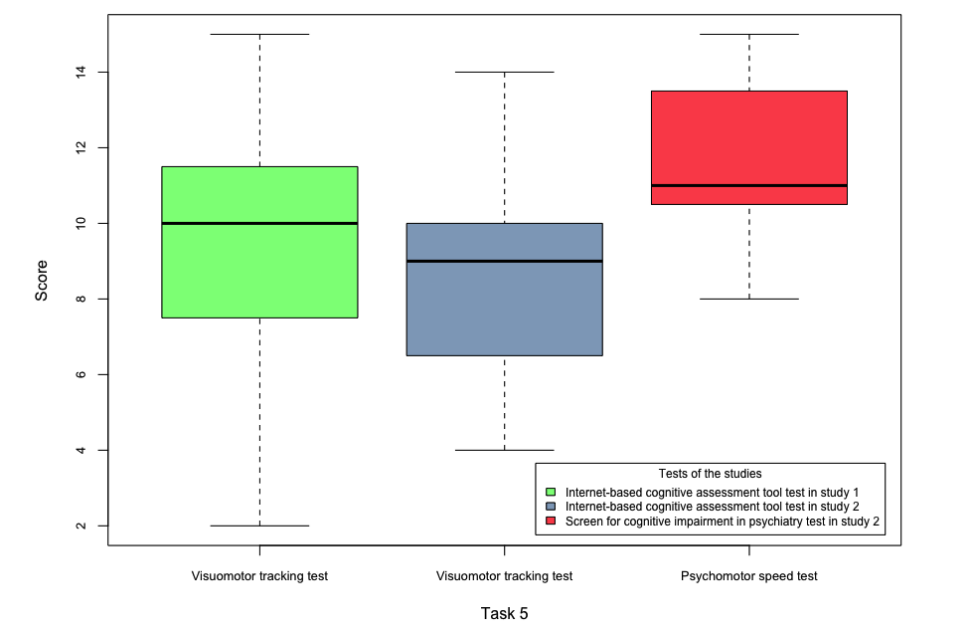
Boxplots of the internet-based cognitive assessment tool and screen for cognitive impairment in psychiatry subscores of the participants of both studies in task 5.

The scores obtained by the participants of both studies in tasks 1-5 are shown in Figures 6-10.

### Usability and Feasibility Outcomes

Of the total number of subjects in both studies (N=40), 37 participants submitted the PSSUQ. The psychometric factors of the PSSUQ results (Table 2) are reported for each study separately because the objectives and procedures of those studies were different. Moreover, the PSSUQ results are calculated for Danish and English test participants. According to the reports collected from the follow-up interview and additional comments received via the PSSUQ form, some of the participants reported some issues and gave some suggestions regarding the instructions and the functionality of the ICAT tests. A total of 2 participants of Study 1 mentioned that there were too many instructions in the ICAT LL task. A participant of Study 1 said that the sorting module in the ICAT CR task was complicated and thus not user friendly, and 2 participants of Study 1 mentioned that this module was problematic. In total, 2 participants of Study 2 mentioned that the ICAT CR task was far easier than the SCIP CR task. A participant of Study 1 suggested replacing some of the textual information in the instructions of the ICAT WAIS LNS task with an example. We did not receive any comment on the ICAT DLL task, perhaps because its functionality was similar to the ICAT LL task. For the ICAT VMT task, a participant of Study 1 mentioned that the time limit of this task was too short. A total of 2 participants of Study 1 mentioned that the practice sets of the ICAT CR, WAIS LNS, and VMT were helpful in understanding the tests.

The results of the correlation analysis between the SCIP-D and ICAT subscores and total scores can be found in Table 3.

**Figure 11:**
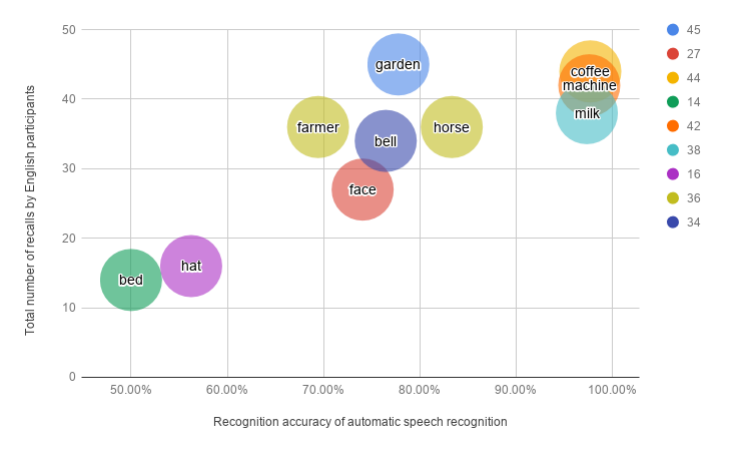
Total number of recalls versus the recognition accuracy of the English words in task 1 (list learning) and task 4 (delayed list learning).

**Figure 12:**
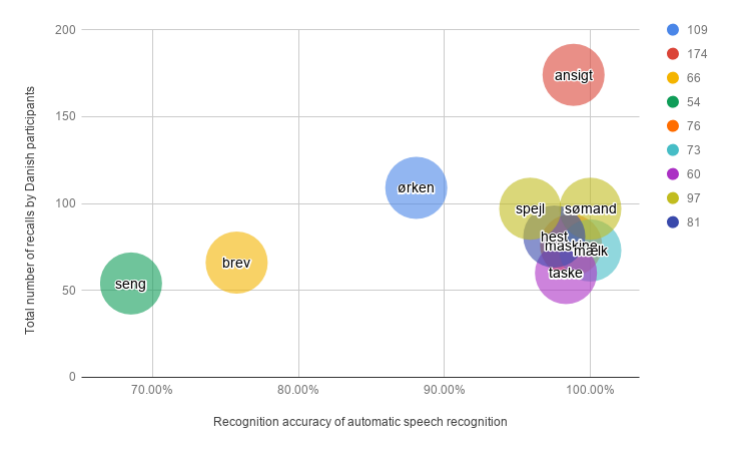
Total number of recalls versus the recognition accuracy of the Danish words in task 1 (list learning) and task 4 (delayed list learning).

The analysis of ASR for the ICAT LL and DLL tasks are reported in Table 4. As can be seen, the insertion (I) rate is 0 for both languages. The number of recalls versus recognition accuracy of each English and Danish word are represented in Figures 11 and 12, respectively. Overall, 332 words were received from 12 English-speaking participants of Study 1 and 887 words were gathered from 28 Danish-speaking subjects (9 from Study 1 and 19 from Study 2). Note that the words which are repeated more than once are included in Figures 11 and 12. Of the English words, *machine*, *milk*, and *coffee* were the most recalled and the least misinterpreted words, whereas *bed* and *hat* were highly misinterpreted and were the least memorized terms. The word *garden* was the most recalled word (45 times) but its accuracy (77.78%) was not as high as the words mentioned earlier. For the Danish word list, *mælk* and *sømand* were correctly recognized for every response received, whereas *seng* and *brev* were misinterpreted frequently.

**Table 2:** Psychometric factors of poststudy system usability questionnaire for usability evaluation of the internet-based cognitive assessment tool reported for both studies and testing languages.

Factor	Study 1 (N=21), mean (SD)	Study 2 (N=16), mean (SD)	Danish test (N=25), mean (SD)	English test (N=12), mean (SD)
Overall score	4.12 (0.46)	4.36 (0.42)	4.25 (0.45)	4.19 (0.45)
System usage	4.23 (0.53)	4.52 (0.41)	4.39 (0.48)	4.35 (0.45)
Information quality	3.86 (0.55)	4.24 (0.58)	4.11 (0.55)	3.84 (0.64)
Interface quality	4.28 (0.62)	4.25 (0.49)	4.16 (0.57)	4.50 (0.45)

**Table 3:** Results of correlation analysis applied to the screen for cognitive impairment in psychiatry (Danish version) and internet-based cognitive assessment tool scores.

Cognitive domain	Screen for cognitive impairment in psychiatry–Danish version task	Internet-based cognitive assessment tool task	Pearson correlation coefficient (r)	*P* value
Verbal learning (SCIP-2^a^)—using ASR^b^ transcripts	VLT^c^-I	LL^d^	0.56	.013
Verbal learning (SCIP-3^e^)—using ASR transcripts	VLT-I	LL	0.67	.002
Verbal learning (SCIP-3)—using manual transcripts	VLT-I	LL	0.66	.002
Working memory (SCIP-2)	WMT^f^	CR^g^	−0.12	.63
Working memory (SCIP-3)	WMT	CR	0.11	.65
Executive function (SCIP-3)	Verbal fluency test	Wechsler adult intelligence letter-number sequencing	0.29	.27
Delayed recall (SCIP-3)—using ASR transcripts	VLT-D^h^	DLL^i^	0.34	.15
Delayed recall (SCIP-3)—using manual transcripts	VLT-D	DLL	0.58	.009
Psychomotor speed (SCIP-3)	VMT^j^	VMT	0.71	.001
Total score	Total	Total	0.63	.009

^a^SCIP-2: Screen for Cognitive Impairment in Psychiatry–version 2.

^b^ASR: automatic speech recognition.

^c^VLT-I: verbal learning test-immediate.

^d^LL: list learning.

^e^SCIP-3: Screen for Cognitive Impairment in Psychiatry–version 3.

^f^WMT: working memory test.

^g^CR: Consonant Repetition.

^h^VLT-D: verbal learning test–delayed.

^i^DLL: delayed list learning.

^j^VMT: visuomotor tracking.

**Table 4:** Performance evaluation of automatic speech recognition in internet-based cognitive assessment tool task 1 (list learning) and task 4 (delayed list learning).

Language	Participants in task 1, n	Participants in task 4, n	Average word error rate	Substitution error ratio, %	Deletion error ratio, %
English	12	11^a^	17.77	77.97	22.03
Danish	28	27^b^	6.31	92.98	7.02

^a^1 English-speaking participant accidentally clicked on the stop button in the internet-based cognitive assessment tool delayed list learning task before repeating the recalled words.

^b^1 Danish participant could not remember any word in the internet-based cognitive assessment tool delayed list learning task.

## Discussion

### Principal Findings

The ICAT is the first Web-based cognitive screening tool for affective disorders, designed based on the SCIP as a gold-standard tool, and it uses ASR to assess immediate and delayed verbal recall. The key findings were that the ICAT was easy to use, had promising feasibility outcomes in measuring key cognitive functions, and had acceptable concurrent validity. Specifically, the ICAT and SCIP-3 total scores correlated to a moderate to strong degree (r=0.63; *P*=.009), and the subtests, namely, LL and VMT, correlated to a moderate (r=0.67; *P*=.002) and strong (r=0.71; *P*=.001) degree, respectively. The usability evaluation of the ICAT system revealed high scores above 4 for system usefulness, interface quality, and overall usage. The information quality was rated lower by the English-speaking participants (3.84), compared with the Danish participants (4.11), which may indicate that the English instructions of the ICAT tests should be revised. The insignificant error rates of ASR, as calculated for the Danish and English responses (6.3% and 17.8%, respectively), indicate a promising future of ASR, particularly for Danish-speaking patients who will be the primary users of the ICAT. According to the results obtained from the recent THINC-it validity study on healthy subjects [10], the 2 cognitive games called Trails (executive function, r=0.74) and Codebreaker (attention, working memory, and executive function, r=0.63) revealed strong to moderate convergent validity, respectively, whereas Symbol Check (working memory, r=0.19) and Spotter (attention, r=0.44) showed low validity. In our study, the ICAT subtest for psychomotor speed also showed moderate concurrent validity, as did the subtest for verbal memory. However, the subtests tapping into working memory and executive skills did not correlate with the original SCIP tasks, which might be because of a suboptimal design of these tests or the small sample size. The ICAT may be an alternative to the THINC-it, which is the most recent cognitive screening tool developed specifically for UD patients. The analysis between the total scores of the SCIP and ICAT showed moderate to strong correlations (r=0.63) in contrast to the moderate concurrent validity (r=0.42) of the THINC-it composite. The higher concurrent validity and the automatic real-time verbal memory assessment via ASR are thus the advantages of the ICAT.

The lack of statistical significance between task 2 of the SCIP and ICAT might be because of the replacement of the oral countdown task with the sorting module because (1) 2 participants in Study 2 mentioned that the ICAT CR subtest was easier compared with the paper-based SCIP CR and (2) participants received high scores in the ICAT CR subtest for both studies (Figure 7), which may indicate a ceiling effect for this task. The insignificant coefficients may indicate that the participants’ cognitive load in the ICAT sorting module was less than the countdown task in the SCIP CR subtest. Hence, the ICAT will require additional modifications before conducting a larger validation study of healthy individuals and patients with affective disorders.

The lack of statistical significance in the DLL task was unexpected because the ASR component was the same for both the ICAT LL and DLL subtests. When doing a poststudy analysis of the recorded data, we found that poor recognition was mainly rooted in 2 factors: (1) the subject did very fast recalls of the words and uttered them right after each other, with no or limited pauses in-between each word or (2) the subject spoke very quietly and far from the microphone. Therefore, the lack of a statistically significant correlation between the ICAT and SCIP DLL tasks might be because of the various ways in which the participants repeated the recalled words. It was previously shown that speech recognition did not perform well for non-native speakers [22], which perhaps justified the higher WER of the English responses for the participants of Study 1 (11 non-native English speakers). The analysis of the ASR of the English-speaking subjects would be more robust if we could recruit more English-speaking participants, especially native speakers. The words which received the lowest accuracy (*bed* and *hat* from the English list and *seng* and *brev* from the Danish list) should be replaced with other words provided in the SCIP manual. The lower ratio of deletion error indicated that ASR received most of the verbal responses in the ICAT LL and DLL subtests.

Digitizing validated paper-and-pencil tests requires effort in prototyping, iterative design, implementation, and evaluation. The ICAT is the first Web-based application designed based on the SCIP as a gold-standard cognitive test battery. Moreover, to our knowledge, none of the existing digital cognitive assessment tools provides a real-time assessment of verbal memory. Taking it all together, the ICAT is a novel digital tool for cognitive assessment. The feasibility of the ICAT reported in this study indicates a promising use for out-of-clinical assessment. The ultimate goal of our research is to introduce the ICAT as a brief cognitive assessment tool for remote administration and the assessment of affective disorder patients.

### Implications for Future Development

On the basis of our observations, the sorting module in the ICAT CR subtest was difficult to use for most of the participants. In addition to this issue, the analysis did not show significant correlations between the SCIP and ICAT CR subtest. Consequently, the sorting module in the ICAT should be redesigned to resemble the SCIP CR task better, for example, with a speech interface, because changing the type of the interface was perhaps the primary reason for the insignificant correlation coefficient.

To mitigate the speech recognition problems, the ICAT should incorporate detailed instructions and tutorials that teach and train users how to speak loudly, clearly, and close to the microphone. Moreover, the speech recognition should be able to detect when users repeat the words too fast or quietly and then instruct them to slow down or speak more clearly. The goal is to enable the ICAT to be administered by the patient, and hence, a strong emphasis should be placed on providing self-explanatory instructions and tutorials to the users.

### Limitations

This is the feasibility study of the ICAT with a limited number of participants. Despite the promising results, there are a set of limitations of the study. First, the evaluation of the ASR for English-speaking participants was limited because of the few number of native English speakers. We did not evaluate the English proficiency of the participants of Study 1 to examine whether or not the ASR recognition error was because of their English proficiency level. Second, the think-aloud method was not practical, especially during the ICAT LL and DLL subtests in which users repeated their recalled words. As cognitive tests demand mental effort, it was hard for the participants to verbalize their thoughts during the test. Hence, an implicit or objective approach for recognizing participants’ interaction with the system throughout the test would be more practical. Third, the nonsignificant coefficients of the executive function and working memory according to Pearson correlation analysis might be because of the modest sample size of Study 2. Fourth, the SCIP VF task and ICAT WAIS LNS task do not translate directly into exactly the same aspect of executive functions. VF performance has been found to correlate with fluid reasoning and shifting aspects of executive function [23], whereas WAIS LNS more specifically measures working memory [24]. It is worth mentioning that currently, Google’s ASR converts any arbitrary word to the closest meaningful word. Hence, the rationale for replacing the VF task with the WAIS LNS task in the ICAT was the possibility of misinterpretation caused by using the ASR technology. Finally, this pilot study included only healthy control participants. The ICAT is intended to be used for cognition screening in patients with mood disorders. On the basis of the preliminary findings from this report, our group is, therefore, in the process of validating a slightly revised version of the ICAT in patients with mood disorders.

### Conclusions

The ICAT is a patient-administered, Web-based tool to screen for cognitive impairment in patients with affective disorders. The results indicate that the ICAT is a good initial step toward building a digital modified cognitive assessment tool based on the SCIP. The high values of the psychometric factors derived from the PSSUQ scores present the ICAT as a usable and useful tool. The use of real-time ASR during the immediate and delayed recall gave a WER of 17.8% and 6.3% for English and Danish responses, respectively. On the basis of the results and insights derived from this study, future optimization and further validation of the ICAT are now warranted.

## Appendix

Multimedia Appendix 1 User experience design of the (ICAT) internet-based cognitive assessment tool.

## Abbreviations

ASR: automatic speech recognition
BD: bipolar disorder
CACHET: Copenhagen Center for Health Technology
CARP: CACHET Research Platform
CNSVS: central nervous system vital sign
CR: consonant repetition
DLL: delayed list learning
GDPR: general data protection regulation
ICAT: internet-based cognitive assessment tool
ISBD: International Society for Bipolar Disorder
LL: list learning
MCI: mild cognitive impairment
mHealth: mobile health
PSSUQ: poststudy system usability questionnaire
SCIP: screen for cognitive impairment in psychiatry
TEAM: technology enabled mental health
UD: unipolar disorder
UI: user interface
UX: user experience
VF: verbal fluency
VMT: visuomotor tracking
WAIS LNS: Wechsler Adult Intelligence Scale letter-number sequencing
WER: word error rate
