# Supplementary material for: The Internet-Based Cognitive Assessment Tool: System Design and Feasibility Study
Source: JMIR Form Res. 2019 Jul 26;3(3):e13898. doi: 10.2196/13898 (PMC6688443; doi:10.2196/13898)
Supplement: Multimedia Appendix 1 [file formative_v3i3e13898_app1.pdf]

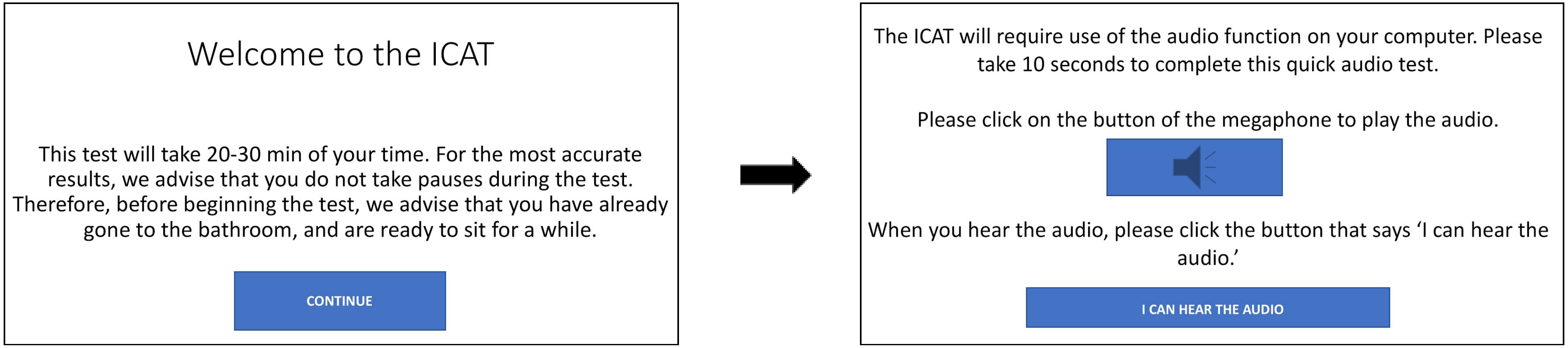

Figure 1: The powerpoint slideshow of the ICAT homepage

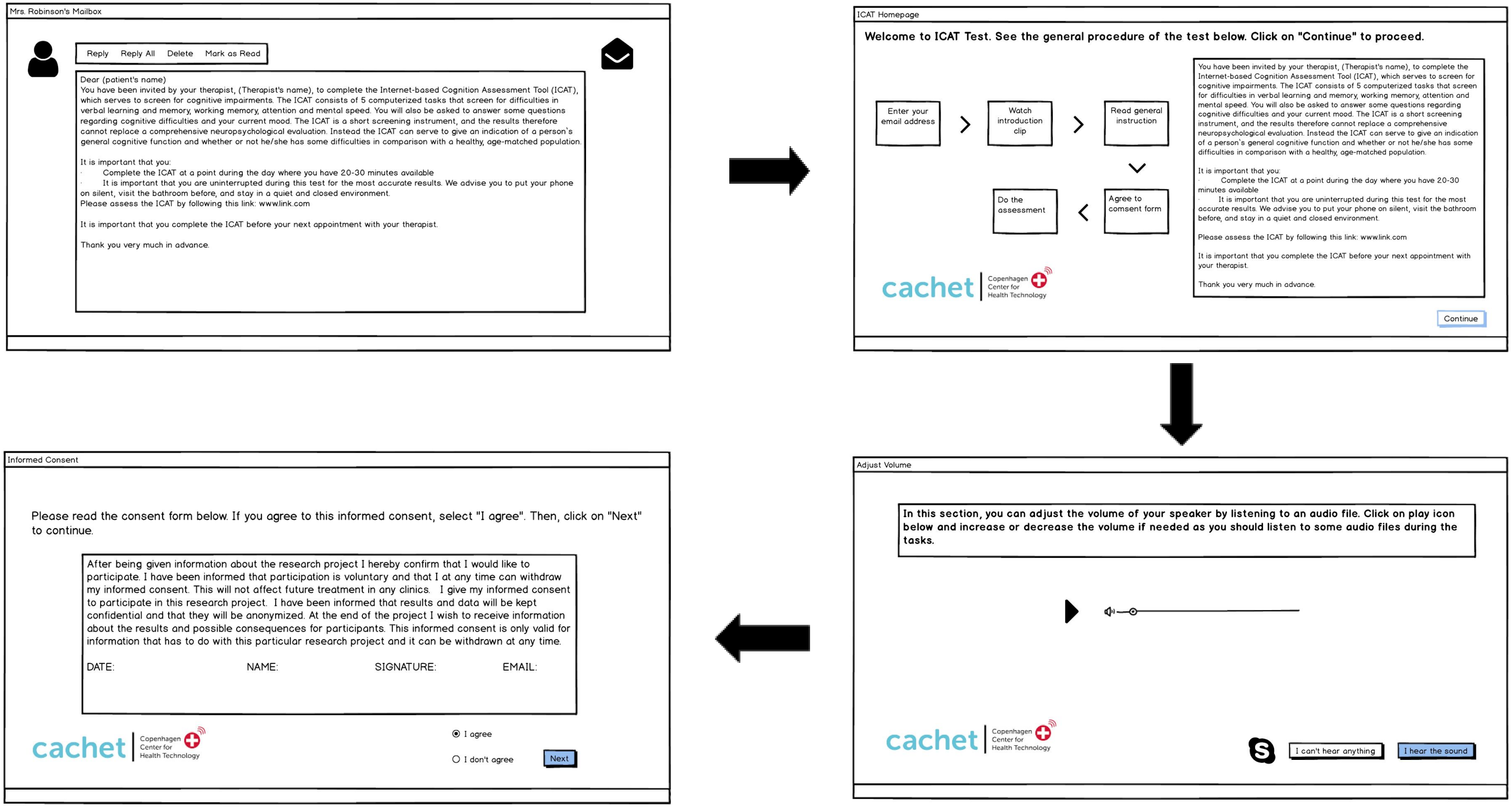

Figure 2: The low-fidelity prototype of the ICAT created in Balsamiq desktop application. Top left: The interface showing how the user is notified to take the ICAT test; Top right: The general tasks of the system as well as the instructions are presented; Bottom right: the user tests the device speaker before beginning the test; Bottom left: consent form

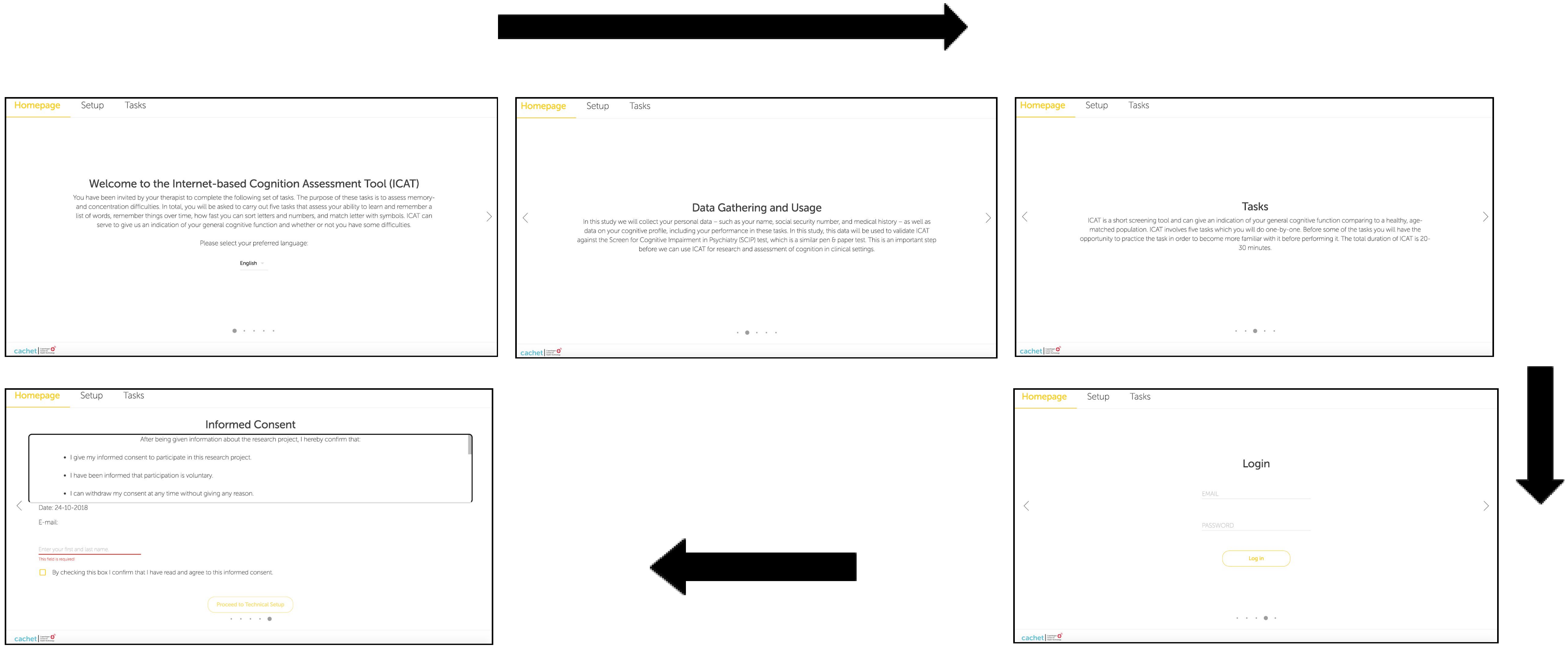

Figure 3: The final design of ICAT homepage before beginning the technical setup. The pagination indicators show the current page.

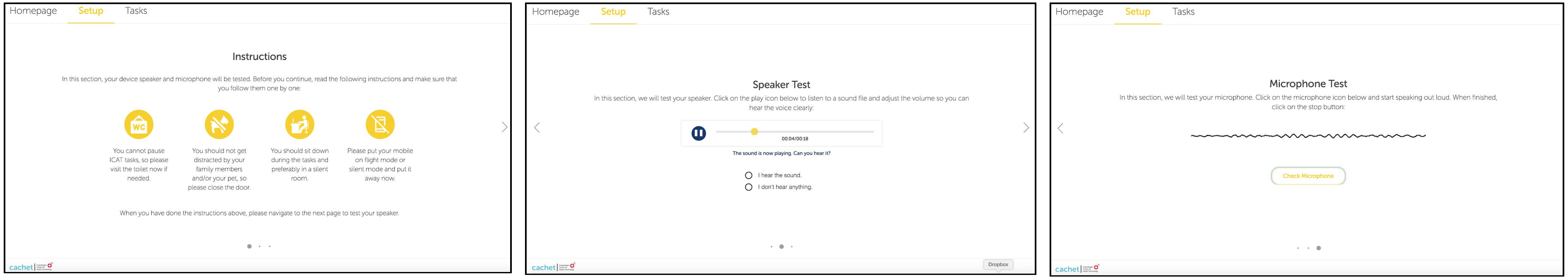

(a) (b) (c)

Figure 4: The final design of ICAT setup including general instructions before beginning the first task. (a) Instructions; (b) Speaker test; (c) Microphone test.
